# Xanthohumol loaded self‐nano emulsifying drug delivery system: Harnessing neuroprotective effects in Alzheimer’s Disease management

**DOI:** 10.1002/alz.087955

**Published:** 2025-01-09

**Authors:** Bushra Bashir, Sachin Kumar Singh, Monica Gulati, Sukriti Vishwas, Kamal Dua

**Affiliations:** ^1^ School of Pharmaceutical Sciences, Lovely Professional University, Jalandhar, Punjab India; ^2^ Faculty of Health, Australian Research Centre in Complementary and Integrative Medicine, University of Technology Sydney, Ultimo, NSW, 2007, NSW Australia; ^3^ Discipline of Pharmacy, Graduate School of Health, University of Technology Sydney, Ultimo, NSW, 2007, NSW Australia

## Abstract

**Background:**

Alzheimer’s disease (AD) is a neurodegenerative disorder characterized by the impairment of cognitive development and disruption of neurocognitive function. This neuropathological condition is marked by neurodegeneration, loss of neural tissue, and the formation of neurofibrillary tangles and Aβ plaques. Various studies reported the utilization of phytoconstituents like fisetin, quercetin, berberine, and xanthohumol for the treatment of AD. Among these phytoconstituents, XH is reported to have neuroprotective effects against AD by attenuating the levels of acetylcholinesterase (AChE) enzyme, accumulated amyloid β, oxidative stress, neuroinflammation, and mitochondrial dysfunction. In addition, XH also acts as senolytic, reducing the impact of cellular aging. Nevertheless, XH exhibits constrained solubility and encounters difficulty in traversing the blood‐brain barrier, thereby diminishing its efficacy in the target sites.

**Method:**

To overcome these challenges, a self‐emulsifying drug delivery system (SNEDDS) for XH was devised, employing quality by design (QbD) based Box‐Behnken design (BBD). SNEDDS offers numerous advantages, encompassing increased drug loading, facile preparation, exceptional stability, as well as enhanced bioavailability and permeability across the blood‐brain barrier.

**Result:**

The optimized XH‐loaded SNEDDS formulation exhibited droplet size, PDI, zeta potential, and drug loading as 40.23nm, 0.2, ‐15Mv, and 98% respectively. The formulation was found to be stable after six months in accelerated stability studies. The formulation underwent various pharmacodynamic studies. Pharmacodynamic studies assessed cognitive and motor functions in rats, revealing significant improvements in cognition with both low and high doses respectively. Additionally, the biochemical investigations revealed that the XH‐loaded SNEDDS effectively decreased the levels AChE enzyme, amyloid β, oxidative stress, and neuroinflammation.

**Conclusion:**

The study demonstrates that the utilization of XH‐loaded SNEDDS for addressing the challenges associated with AD treatment. The optimized XH‐loaded SNEDDS formulation exhibited favorable characteristics and stability, overcoming XH solubility constraints. The results suggest that the SNEDDS approach enhances the bioavailability and permeability of XH, highlighting its potential as a neuroprotective and senolytic agent for AD treatment.

